# Experience on the management of the first imported Ebola virus disease case in Senegal

**DOI:** 10.11694/pamj.supp.2015.22.1.6109

**Published:** 2015-10-10

**Authors:** Bousso Abdoulaye, Seydi Moussa, Ka Daye, Badiane Seydou Boubakar, Sarr Samba Cor, Talla Idrissa, Ndiaye El Hadj Mamadou, Ba Ibrahima Oumar, Ndour Cheikh Tidiane, Ly Mamadou Selly, Diop Cheikh Tacko, Diack Papa Amadou, Loume Mandiaye, Diouf Mbaye, Coll-Seck Awa Marie

**Affiliations:** 1 Ministry of Health, Senegal; 2 Fann National Hospital, Dakar, Senegal

**Keywords:** Ebola, imported case, Senegal, managment

## Abstract

The Ebola virus disease, as a first epidemic in West Africa, stands as the most deadly one throughout history. Guinea, the source of the epidemic, Sierra Leone and Liberia remain the most strongly affected. That epidemic thoroughly destabilized the health system of those countries. Following Nigeria, Senegal received its first imported case from the neighboring Republic of Guinea. In that sub regional psychotic context, such a situation has been handled and managed starting from the potential of a health system that is already suitably structured. The organization of the response, the management of the communication system and the rigorous monitoring of contacts have been decisive in the control of the epidemic. Our countries have to be prepared in order to face health threats, and that is the reason why the need to empower our health systems is important.

## Introduction

Since the official declaration of the existence of the Ebola virus disease outbreak in Guinea by the World Health Organization (WHO) on March 23, 2014 [1], all West African countries were under jeopardy. Liberia, on March 13 [2] and Sierra Leone, on May 28 [3] will be the two other seriously affected countries. Nigeria, on July 20 will be the fourth affected country subsequent to the importation of a case from Liberia [4]. On August 26, the authorities of the Democratic Republic of Congo declared their first cases [5]. Senegal, the sixth affected country, will confirm its first case imported from Guinea on August 29. The United States of America, the seventh affected country will diagnose their first imported case from Liberia on September 30 [6]. Senegal, Nigeria and the United States share the specificity of having cases that are said to be imported, by road way for the first case and by air flight for the two others. The diagnosis of the first Ebola virus case imported in Senegal was a test for our healthcare system.

## Patient and observation

On August 29, 2014, The Minister of Health officially announced the First Ebola virus disease case in Senegal. It was related to a 21-year-old national from Guinea, who had been in contact in Guinea with members of his family who had contracted the disease and eventually passed away. He entered Senegal by road, before the second restricted access of frontiers on August 22. He had used an overnight public transport vehicle from August 13 through 14 in order to reach Dakar. At that time, and all along his trip, he had presented no single fever-type symptom. Three days after his arrival, he started showing signs of fever, vomiting and diarrhea with no bleeding, and then decided to consult in a suburban health centre where he had been taken in charge for three days. Due to persisting symptoms, he approached the infectious diseases service at Fann University Hospital, where he was subsequently hospitalized. During all those consultations, he had never informed practitioners as regards the contacts he likely had with other people affected by the Ebola virus disease. Senegal had been alerted by some WHO authorities based in Guinea that a contact-case had probably travelled to Senegal; the investigation undertaken revealed that it was related to the patient hospitalized at the Fann Hospital Infectious Disease Service. He was then immediately placed in an isolation room and some fluid samples were gathered. On August 28, the test operated on the blood sample by the Dakar Pasteur Institute returned as Zaire-Ebola-virus-positive. The investigations undertaken helped identify 74 individuals who had been in contact with the patient (34 in his family and 40 health workers). The reopening of the interrogation of both the patient and his caretaker, with a focus on their itinerary, facilitated the identification of all the family members and the health centre staff. At the level of the hospital, the patient's record was reviewed, along with the list of all personnel on duty. This helped identify all individuals who had been in contact with the patient or his biological samples. All the contacts were put in quarantine inside their homes and were monitored twice a day by the medical district teams in their respective areas, conducting temperature checks and patient questioning. Senegal, since the declaration of the outbreak in Guinea, had implemented a three-step response plan: pre-epidemic, epidemic and post-epidemic. Since the month of March, the National Committee for Epidemics Administration has held weekly meetings in order to implement the pre-epidemic phase activities. This is a permanent committee meeting anytime a threatening epidemic situation occurs in Senegal.

During the pre-epidemic phase, all activities essentially deal with the communication of health workers to populations, the training of healthcare personnel, the capacity-building of healthcare workers and the reinforcement of frontiers surveillance. As soon as the case was signified, the epidemic phase was launched and ten commissions were implemented adding to the National Committee for Epidemics Management. A coordination committee supervised by the Healthcare Executive Director including commission presidents and their reporters, along with some partners would meet every morning. The multi-sectorial approach was used as a guide for the commissions’ implementation. Epidemiological Investigation and Surveillance Commission: in charge of the monitoring of the 74 contact cases and alert management; social Intervention and Behavior Commission: in charge of the patient's and contacts’ psychosocial care; media and Communication Commission: in charge of all aspects dealing with communication; patients’ Clinic Care and Infection Control Commission: in charge of the monitoring of the patient's care and also the supervision of healthcare structures; logistics Commission: in charge of the management of all necessary products, facilities and material; security Commission: in charge of patients and contacts security and frontier surveillance; research and ethical aspects commission: in charge of all research and ethical issues related to the epidemic: hygiene and Sanitation Commission: in charge of the decontamination and sanitation healthcare sites; fund-raising Commission: in charge financial resources management. Mobile Intervention Commission: in charge of the transportation and the sampling of all suspect cases.

## Discussion

That first Ebola virus case allowed our services to control and assess the reaction capacity of our healthcare system to efficiently handle situations of healthcare emergencies. The control of any epidemic requires a well-organized healthcare system [7, 8]. Senegal had to manage some outbreaks such as cholera [9] or meningitis [10], but this was the first time the country had to face a highly lethal pathology that had such a strong social and economic impact.

**The patient:** during his various consultations the patient had never declared the type of contacts he had had with other affected people. The different interrogations conducted subsequent to hospitalization contributed to the collection of new elements that had not been initially mentioned. That notion of information retention stands as a noteworthy fact which is shared with the case imported from Nigeria [4]. Even if the patient never revealed some likely contact with affected people, the absence of bloody diarrhea hindered the initial diagnosis. That was a compelling sign in our case definition process, such as specified by the WHO [11]. Following that experience, we were bound to modify our case definition process, while making it clear that hemorrhage was not always present in suspect cases diarrhea. That imported case raised prevailing indignation among the national public opinion, ending in the stigmatization of Guinean populations living in the country. The patient was completely healed after a 23-day hospitalization.

**Contacts monitoring:** as a whole, 74 contacts have been monitored twice a day for 21 days. Over the first 15 days, 67 people had been identified. On day fifteen, 7 new contacts and healthcare workers identified themselves after they had been trained about Ebola. Those individuals agreed then that they responded to the definition of contact. All the individuals, amounting to 34, that had shared the patient's home, had been confined in that place, subsequent to its decontamination. Their food, along with their security, was guaranteed by the Government. The rest was composed by the healthcare personnel (medical doctors, nurses, laboratory technicians and orderlies), whose monitoring in the beginning was harder to handle, since some of them were reluctant to allow the agents in charge of their monitoring, because they did not want the members of their families to be informed of their situation as contacts. After a 21-day monitoring process, all the contacts were declared healthy, despite a few alerts during the follow-up period.

**Lessons learned:** in order to face an outbreak such as the Ebola virus disease, it is important to basically benefit from a well-organized healthcare system. The multi-sector approach is also highly decisive in the implementation of strategies, since the healthcare authorities alone are unable to settle all issues. At higher level, the state's commitment is extremely important in order to face such a plague. The technical support of partners (WHO, UNICEF, CDC and MSF) was highly appreciated in the organization's restructuring and the strategies implemented. The support of experts who have the benefit of some hindsight as regards the country's situation allows to objectively refine any response plan. Our organization system, which is structured into commissions, facilitated the implication of all actors and sectors concerned. However, at certain times, such organization seemed very heavy to monitor, due to some issues dealing with decision-making coordination and priority. Even though the pertinence of the commissions’ implementation is not really questioned, the best approach would be to have a single coordination body that will supervise all the tasks of those commissions. That body could be in the form of some emergency operations center, as decided in our country, following the example of the situation drawn from Nigeria [4] or other countries. The main aspect that needs be avoided during those moments: to transform the Ministry of Health into a Ministry for Ebola and thus paralyze all other health programs. For instance, in Guinea, Liberia and Sierra Leone, all programs against malaria have been seriously compromised [12]. The behavior of health workers has been exemplary even though some stress or panic situation has sometimes been observed. It is important to consider that some attitudes can be unpredictable in face of a real case even if the person had been theoretically well-trained. The presence and commitment of highly-qualified healthcare professionals help reassure the personnel, and consequently obtain their adhesion in the handling of cases. The monitoring of healthcare professional contact cases is at times difficult to handle. They often prove some resistance, either because it is not easy for them to accept their own situation, or they are able to somatise very quickly. It is imperative for those people to benefit from accurate psychological support. The contribution of their line manager, as regards this support, is crucial in order to obtain their adhesion. The management of all communication directed to populations stood as the most sensitive aspect. Even if public-awareness campaigns have been organized before the epidemic, the manifestation of one single case can definitely alter all information. A situation of panic can quickly settle down and in our case, the stigmatization of a whole community was the most sensitive aspect observed. The commitment of journalists as regards the responsible management of the information during the crisis could be obtained after a meeting session with the Minister of Health and all media editors. The Minister took that opportunity to sensitize journalists on the negative impact of non verified information on the society and the country more generally, and also she promised to share with them any necessary information. The healing and return of the patient in his native country helped, in some way, alleviate the social tension it had caused. The involvement of the Minister of Health, from the beginning of the communication process, while committing herself to provide all existing information, has been noted and highly appreciated. The option to set up press releases and conferences supervised by the Minister herself, based on the necessity of the moment, was glorified and acknowledged as a decisive strategy to fight the epidemic away.

## Conclusion

The Ebola virus disease is an affection that challenges our healthcare systems. Our states’ main objective should be to build up a performing healthcare system that can help face all sorts of threats. The experience of Senegal demonstrated the fact that African countries can also benefit from a well-organized healthcare system and competent personnel who have the capacity to handle the most serious epidemic threats. The implementation of an Emergency Operations Center in our countries should also stand as a priority in order to benefit from efficient and well-coordinated task and reaction forces.
